# Impact of a very low‐calorie ketogenic diet on metabolic and microbiota outcomes in post‐bariatric patients and bariatric‐Naïve individuals: A comparative pilot study

**DOI:** 10.1111/dom.16187

**Published:** 2025-01-10

**Authors:** Ilaria Ernesti, Maria Chiara Massari, Fiammetta Cipriani, Davide Masi, Krzysztof Glaser, Martina Genco, Dario Tuccinardi, Carla Lubrano, Stefania Mariani, Antonio Angeloni, Lucio Gnessi, Sabrina Basciani, Mikiko Watanabe

**Affiliations:** ^1^ Department of Surgical Sciences Sapienza University Rome Italy; ^2^ Section of Medical Pathophysiology, Food Science and Endocrinology, Department of Experimental Medicine Sapienza University Rome Italy; ^3^ Department of Endocrinology Metabolism and Internal Medicine Poznan University of Medical Sciences Poznań Poland; ^4^ Unicamillus‐Saint Camillus International University of Health Sciences Rome Italy; ^5^ Research Unit of Endocrinology and Diabetology, Department of Medicine and Surgery Università Campus Bio‐Medico Rome Italy; ^6^ Fondazione Policlinico Universitario Campus Bio‐Medico Rome Italy; ^7^ Department of Experimental Medicine Sapienza University Rome Italy

**Keywords:** bariatric surgery, dietary intervention, obesity care, observational study, weight control, weight management

## Abstract

**Aims:**

To date, bariatric surgery (BS) is the most effective long‐term treatment for obesity, but weight regain (WR) is common. The very low‐calorie ketogenic diet (VLCKD) is effective for weight loss and may influence gut microbiota (GM) composition, but it has been scarcely evaluated in post‐bariatric patients. This study compared the efficacy and safety of a VLCKD in patients with WR post‐bariatric surgery (BS+) and in bariatric surgery‐naïve patients (BS‐).

**Methods:**

In this prospective, case–control study, 33 patients (15 BS+, 18 BS‐) underwent an 8‐week‐long VLCKD. Outcomes included weight loss, metabolic profile, safety and GM composition.

**Results:**

Both groups achieved significant weight loss (BS+: −6.9%, BS‐: −8.3%), but the BS+ group showed slightly less metabolic improvement, particularly in insulin resistance and triglycerides. GM composition differed at baseline, reflecting the lasting effects of BS, and VLCKD led to significant changes in both groups. Microbial diversity and specific taxonomic shifts were more pronounced in BS‐ patients. Mild renal function changes were noted in BS+ patients, though these remained within clinically acceptable ranges.

**Conclusion:**

VLCKD is effective in both BS+ and BS‐ patients, though metabolic and microbial responses may be less robust post‐surgery, possibly due to anatomical and physiological changes. Tailored approaches may be therefore needed to optimize outcomes in post‐bariatric patients.

## 1 INTRODUCTION

Obesity has reached pandemic dimensions,^1^, ^2^ with its pathophysiology becoming increasingly understood, though much remains to be uncovered. Among others, obesity is linked to gut microbiome (GM) dysbiosis, characterized by alterations in microbial composition, richness and function contributing to metabolic dysfunctions and energy balance disruption.^3^

Bariatric surgery (BS) is the most effective long‐term treatment for obesity,^4^, ^5^ inducing anatomical changes that alter hormone levels, increase satiety and reduce hunger.^6^ BS derives changes in dietary preferences, nutrient absorption, bile acid metabolism and gut hormone production^7^, ^8^, ^9^; enhances microbial diversity and richness^7^, ^8^, ^10^; increases beneficial bacteria^10^, ^11^; and reduces harmful ones,^7^, ^10^ contributing to improved metabolism and health.^8^, ^9^

Post‐bariatric weight regain (WR) is a common challenge, often linked to the reversal of hormonal changes,^12^ hypoglycaemia^13^ and maladaptive behaviors.^12^ Metabolic adaptation, the difference between actual and expected energy expenditure after weight loss, is also key.^14^ The GM may also drive WR, with distinct GM composition observed in those experiencing WR,^15^ and longitudinal data suggesting a role in determining post‐surgery weight trajectory.^16^ WR frequently reintroduces obesity‐related complications, reduces quality of life and increases healthcare costs. Effective treatments are needed, as no lifestyle intervention has shown significant benefit.^17^

The very low‐calorie ketogenic diet (VLCKD), characterized by a very low calorie and carbohydrate intake and the use of protein supplements, is one of the pivotal approaches for the treatment of obesity.^18^, ^19^, ^20^, ^21^, ^22^ Noteworthy, the GM is a dynamic system influenced by diet, and VLCKDs seem to positively affect GM composition and diversity.^23^ Literature on the effects of VLCKDs in post‐BS patients is limited to two studies, suggesting benefits but not exploring GM changes.^24^, ^25^ Additionally, no study has ever compared the efficacy and safety of VLCKDs on post‐bariatric patients versus those who are BS‐naïve. Considering the distinct physiology and anatomy of post‐bariatric patients, we hypothesized that the benefits of VLCKDs might differ in this population, and our primary outcome was to evaluate these potential differences. Secondary objectives included assessing changes in metabolism, body composition and the GM.

## 2 MATERIALS AND METHODS

### 2.1 Study design and population

This was a single centre, prospective, case–control, pilot study investigating the safety and efficacy of a VLCKD in patients with post‐bariatric WR, compared to BS‐naïve. This 8‐week‐long study assessed safety, efficacy and dietary compliance. Participants were recruited from the high‐specialization obesity treatment centre (CASCO) at Sapienza University, Rome. Eligibility criteria included individuals aged 18–65 years with a body mass index (BMI) ≥30 kg/m^2^. For the case group (BS+), participants required a history of Roux‐en‐Y Gastric Bypass (RYGB) or vertical sleeve gastrectomy (VSG), with clinically significant WR following an initial weight loss of ≥20% of total body weight. Clinically significant WR was defined as regaining more than 10% of the maximum weight lost after surgery.^26^ Exclusion criteria included: type 1 diabetes, GFR <60 ml/min/1.73 m^2^, decompensated cirrhosis, congenital metabolic diseases, pregnancy or lactation, major psychiatric disorder, alcohol and drug addiction, no self‐sufficiency in the absence of adequate support. Cases and controls were matched by BMI, age and sex, with a minimum female‐to‐male ratio of 2:1, ensuring representation of males while accounting for the higher female prevalence in this population. Demographic data were collected via structured interviews. The study was approved by the local IRB (ref. 5475) and conducted in accordance with the Declaration of Helsinki and Good Clinical Practice. Written informed consent was obtained before enrollment. The trial was registered on Clinicaltrials.gov (NCT05896358).

### 2.2 Dietary treatment

All patients underwent a VLCKD regimen, with a carbohydrate intake <50 g/day, protein intake 1.2–1.5 g/kg of ideal body weight, and fat making up for the rest of the caloric intake. Energy requirements were calculated based on total energy expenditure, derived by multiplying basal metabolic rate by physical activity level. During the first 4 weeks, patients consumed protein supplements along with one very low carbohydrate meal per day (e.g., meat, fish, eggs or cheese), with low glycaemic index vegetables, and a calorie deficit of ~600 kcal/day. The following 4 weeks, another protein supplement was replaced with a very low carbohydrate meal, with a deficit of ~300 kcal/day, and slight calorie intake variations depending on meal choices. Participants were to drink 2 L of water/day and to select vegetarian and healthy sources of fat; with protein mainly coming from fish, eggs, fresh dairy products and lean meat. The potential micronutrient intake deficiency was addressed through recommended vitamins, minerals and omega‐3 supplements.^27^ Compliance was monitored through 4‐weekly dietician consultations, 3‐day dietary recalls and capillary beta hydroxybutyrate (BHB) levels, a ketone produced from acetyl‐CoA and beta‐oxidation of fatty acids in the liver. This is a reliable biomarker for dietary adherence since it reflects nutritional ketosis.

### 2.3 Biochemical measures

Biochemical tests followed standard procedures using blood samples collected after an 8‐h overnight fast. These included electrolytes, glucose, insulin, lipid profile (triglycerides and total, high‐density lipoprotein [HDL‐c] and low‐density lipoprotein [LDL‐c] cholesterol), creatinine, alanine aminotransferase (ALT), aspartate aminotransaminase (AST), uric acid and estimated glomerular filtration rate (eGFR). BHB was measured in duplicate using enzyme colorimetric assays (StanBio, Boerne, TX).

### 2.4 Anthropometric and body composition assessment

Body weight was measured using a balance‐beam scale (Seca GmbH & Co, Hamburg, Germany) under standardized conditions: fasting, barefoot, wearing only light clothing and with an empty bladder. Height was rounded to the closest 0.5 cm. Waist circumference (WC) was measured midway between the lower rib and the iliac crest, hip circumference (HC) at the level of the widest circumference over the great trochanters to the closest 1.0 cm. Body composition was measured by Dual X‐Ray Absorptiometry (DXA) (QDR Discovery Acclaim, Hologic Inc., Waltham, MA) as previously described.^28^ Blood pressure (BP) was measured with an automated digital device. Patients on antihypertensives were advised to contact the team in case of BP reduction.

### 2.5 Adverse events

The occurrence of nausea, vomiting, reflux, bloating, belching, abdominal pain, constipation, diarrhoea and fatigue were recorded at each visit through a structured questionnaire including timing, duration and entity.

### 2.6 Stool collection and GM taxonomic composition

Stool samples were collected using the DNA Shield Fecal Collection Tube with beads. Participants collected samples at home following standardized instructions and returned them under proper storage conditions. DNA was extracted using the Qiacube HT with the Qiagen DNeasy 96 PowerSoil Pro Kit from a subset of both groups (7 vs. 6 patients), as some patients did not agree to stool collection while others brought inappropriately collected samples and were excluded. Briefly, 50–100 mg of faecal material was lysed with zirconium beads and lysis solution (CD1) at 76°C for 15 min, followed by mechanical disruption using the Tissue Lyser at 25 Hz for 10 min. The lysate was centrifuged, and the supernatant was processed for DNA extraction. Sequencing and bioinformatics pipeline followed standard protocols described previously,^23^ key differences included the use of modified primers^29^ and stricter quality filtering during denoising. Briefly, the bioinformatics analysis was conducted using QIIME2 (version 2023.7), with the DADA2 plugin employed for denoising. Forward and reverse reads were truncated at 270 and 215 bp, respectively, with quality filtering applied using the default truncQ cutoff and a maximum expected error (maxEE) of 2 for both forward and reverse reads. Sequences were dereplicated and chimera‐checked. Primer removal was performed using Cutadapt (version 2023.7). Additional filtering steps were applied, including length >350 bases and frequency >0.01%, resulting in 647 high‐quality ASVs. Taxonomic assignments were conducted using the GreenGenes 2 (version 2022.10) and Silva (version 138) databases. To summarize, visualize and interpret metagenomic data, several key metrics and indexes were used, including metagenomic richness, counting the genes identified in a sample; alpha diversity, measuring different species variety and abundance within a sample; beta diversity, examining species differences between samples; and the relative abundance of bacteria at different taxonomic levels.

### 2.7 Statistical analysis

Statistical analysis was performed using SPSS Armonk, NY, IBM corp. Ver. 27.0 for Mac. For data visualization and statistical analysis of metadata, (V.1.2) R package was utilized. Descriptive statistics for continuous variables were presented as number, mean, standard deviation, while categorical variables were presented as frequencies and percentages. A general linear repeated measures model, with terms for treatment, time, time*treatment interaction, was used to analyse continuous endpoints. The time*treatment interaction analyses were used to determine groupwise differences. To assess within group differences, we employed the Wilcoxon signed‐rank test for paired samples, and the Pearson *χ* ^2^ test was used for categorical variables. GM alpha diversity was assessed using the observed features index and Faith's Phylogenetic Diversity, with statistical comparisons performed using the Kruskal–Wallis test. Beta diversity was analysed using the Bray–Curtis and Jaccard distance metrics, and comparisons were evaluated using PERMANOVA. Differential abundance was analysed using ANCOM and ANCOM‐BC, and further explored using ANCOM‐BC2 for repeated measures. Changes in GM composition were reported as log‐fold changes in relative abundance.

Differences, associations and interactions were considered significant if *p* < 0.05. The estimated mean weight of the study population based on our data is 110 ± 12 kg. Expecting a 5% BMI loss as clinically relevant, we estimated that a total of 13 patients per group was needed to detect this reduction, with an *α* of 0.05 and a (1‐β) of 80%.

## 3 RESULTS

Thirty‐three patients were initially enrolled. As two patients from the BS+ group withdrew due to difficulties adhering to the diet, the final analysis included 31 patients (completers): 13 patients with (BS+, 9 female, 5 male) and 18 patients without history of BS (BS‐, 13 female, 5 male). Among the BS+ group, the average body weight before BS was 139 ± 25 kg, nadir 87 ± 20 kg, maximal weight loss 51 ± 17 kg and WR of 21 ± 9 kg over time. The timespan between BS and the beginning of the study was 3–18 years, averaging at 7 years. Two patients had a RYGB and eleven had a VSG. Baseline characteristics are summarized in Table 1. All participants had BMI ≥30 kg/m^2^.

**TABLE 1:** Baseline characteristics of study population at baseline.

	BS+	BS‐	*p*
Parameters (UM)	Mean ± SD	Mean ± SD	
N	13	18	
Age (years)	52.5 ± 9.38	50.8 ± 9.98	0.645
Gender(%female)	69.0	72.0	0.980
Body weight (kg)	111.6 ± 23.88	112.2 ± 26.62	0.936
BMI (kg/m2)	41.1 ± 6.54	40.1 ± 6.18	0.660
Waist circumference (cm)	120.8 ± 18.66	118.9 ± 20.84	0.645
Hip circumference (cm)	128.5 ± 10.11	127.3 ± 9.87	0.827
Body fat (%)	41.2 ± 5.72	40.3 ± 5.86	0.660
Lean mass (%)	58.8 ± 5.72	56.3 ± 6.02	0.368
Fat/lean ratio	0.7 ± 0.18	0.7 ± 0.17	0.779
WHR	0.9 ± 0.12	0.9 ± 0.11	0.126
HOMA‐IR	3.0 ± 1.66	4.2 ± 2.71	0.161
Creatinine (mg/dL)	0.8 ± 0.16	0.7 ± 0.18	0.377
GFR (mL/min/1.73m^2^)	94.0 ± 20.00	94.0 ± 15.00	0.965
Blood urea nitrogen (mg/dL)	35.1 ± 10.01	38.8 ± 10.24	0.441
AST (IU/L)	18.4 ± 3.17	20.9 ± 5.38	0.082
ALT (IU/L)	18.9 ± 6.79	26.9 ± 9.90	**0.036**
K (mEq/L)	4.5 ± 0.47	4.2 ± 0.33	0.187
Na (mEq/L)	142.8 ± 4.10	142.2 ± 2.51	0.819
Hba1c (%)	5.4 ± 0.45	5.6 ± 0.49	0.189
Uric acid (mg/dL)	6.0 ± 1.72	5.3 ± 1.18	0.325
Total cholesterol (mg/dL)	181.2 ± 40.29	209.1 ± 38.83	0.068
HDL cholesterol (mg/dL)	58.3 ± 14.11	54.3 ± 14.03	0.534
Triglycerides (mg/dL)	93.7 ± 36.22	122.6 ± 40.94	**0.045**
LDL cholesterol (mg/dL)	104.1 ± 35.17	129.7 ± 34.95	**0.041**

*Note*: Data are presented as mean ± standard deviation (SD). *p* values were obtained using a Wilcoxon test.

Abbreviations: ALT, alanine aminotransferase; AST, aspartate aminotransferase; BMI, body mass index; GFR, glomerular filtration rate; HOMA‐IR, homeostasis model assessment of insulin resistance; WHR, waist‐to‐hip ratio.

In the BS+ group, VLCKD treatment resulted in weight loss of 6.9 ± 3.5% (*p* < 0.001) and a decreased BMI of 5.0 ± 6.7% (*p* = 0.034). The change in WC was 8.2 ± 4.0% (*p* = 0.001). In the BS‐ group, the VLCKD resulted in weight loss of 8.3 ± 2.8% (*p* = 0.002) and a decreased BMI of 8.3 ± 2.7% (*p* < 0.001). The decrease in WC was 7.5 ± 4.3% (*p* < 0.001). Percentage changes in the two study groups are summarized in Table 2. Notably, the BS+ group lost somewhat less weight, but the difference compared to BS‐ did not reach statistical significance (%BMI change *p* = 0.089). Improvements in body composition were evident, as demonstrated by reduced body fat (both *p* = 0.001) and improved waist‐to‐hip ratio (*p* = 0.005 and 0.030, respectively) with no groupwise difference (*p* = 0.29, *p* = 0.34).

**TABLE 2:** Percentage changes in clinical and metabolic parameters from baseline to 8 weeks in both the BS+ and BS‐ groups. p1 represents the paired Wilcoxon test within the BS+ group, p2 represents the paired Wilcoxon test within the BS‐ group and p3 is derived from a repeated measures general mixed model comparing both groups (time*treatment interaction).

Parameters	BS+	BS‐	p1	p2	p3
	Mean ± SD	Mean ± SD			
Body weight	‐6.9% ± 3.5%	−8.3% ± 2.8%	**0.000**	**0.002**	0.274
BMI	−5.0% ± 6.7%	−8.3% ± 2.7%	**0.034**	**0.000**	0.089
Waist circumference	−8.2% ± 4.0%	−7.5% ± 4.3%	**0.001**	**0.000**	0.631
Hipcircumference	−4.2% ± 4.6%	−4.6% ± 1.9%	**0.022**	**0.000**	0.85
Body fat	−6.5% ± 6.7%	−4.6% ± 4.3%	**0.001**	**0.001**	0.37
Lean mass	4.4% ± 4.6%	6.4% ± 11.6%	**0.001**	**0.001**	0.689
Fat/lean ratio	−10.1% ± 9.6%	−4.1% ± 46.1%	**0.001**	**0.016**	0.692
WHR	−4.0% ± 5.9%	−2.3% ± 3.4%	**0.005**	**0.030**	0.346
HOMA‐IR	−27.3% ± 44.4%	−51.2% ± 35.2%	**0.019**	**0.003**	0.093
Creatinine	8.8% ± 5.9%	1.5% ± 13.1%	**0.003**	0.720	**0.046**
GFR	−7.7% ± 5.0%	−0.7% ± 7.0%	**0.003**	0.574	**0.005**
Blood urea nitrogen	26.0% ± 19.8%	7.8% ± 29.9%	**0.001**	0.401	0.182
AST	15.6% ± 23.2%	5.2% ± 29.6%	0.073	0.775	0.17
ALT	7.2% ± 32.4%	−4.1% ± 44.7%	0.562	0.089	0.203
K	−1.0% ± 14.1%	−6.1% ± 8.6%	0.606	0.197	0.495
Na	−1.1% ± 2.4%	−1.3% ± 1.6%	0.160	0.102	0.949
Hba1c	−0.5% ± 7.5%	−5.0% ± 4.0%	0.798	**0.001**	0.061
Uric acid	5.0% ± 10.8%	14.1% ± 30.8%	0.248	0.116	0.611
Total cholesterol	−11.3% ± 13.8%	−12.9% ± 16.4%	**0.013**	**0.002**	0.491
HDL cholesterol	−9.6% ± 10.4%	−4.9% ± 20.8%	**0.015**	**0.047**	0.405
Triglycerides	−14.8% ± 29.9%	−32.2% ± 22.0%	**0.028**	**0.000**	**0.047**
LDL cholesterol	−10.3% ± 18.4%	−7.6% ± 38.5%	**0.039**	**0.010**	0.658

Abbreviations: ALT, alanine aminotransferase; AST, aspartate aminotransferase; BMI, body mass index; GFR, glomerular filtration rate; HOMA‐IR, homeostasis model assessment of insulin resistance; WHR, waist‐to‐hip ratio.

HbA1c was reduced in the BS‐ group (*p* = 0.001), but not in the BS+ group (*p* = 0.798), with similar baseline values (5.6 ± 0.49 vs. 5.4 ± 0.45%, respectively, *p* = 0.189). Similarly, the Homeostatic Model Assessment of Insulin Resistance (HOMA‐IR) was significantly reduced in the BS– group (*p* = 0.003) and in the BS+ group, although with a markedly smaller improvement (*p* = 0.01). There were striking improvements in blood lipid levels in both groups with no significant difference groupwise beyond triglycerides which were significantly lower in the BS‐ group at follow‐up (groupwise *p* = 0.047) (Table 2). Ketosis, reflected by BHB ≥ 0.5 mmol/L, was obtained in 38.5% and 64.7% of the BS+ and BS‐ patients, respectively (*p* = 0.128). This was used as a surrogate compliance indicator.

Regarding safety, there was a small increase in creatinine in the BS+ group (*p* = 0.003), coupled with increased blood urea nitrogen (*p* = 0.001) and decreased eGFR (*p* = 0.003), not present in the BS‐ group, with a significant groupwise difference (creatinine *p* = 0.046, eGFR *p* = 0.005). All other safety parameters were unchanged (Table 2). No adverse events were recorded beyond mild constipation (data not shown).

**FIGURE 1:**
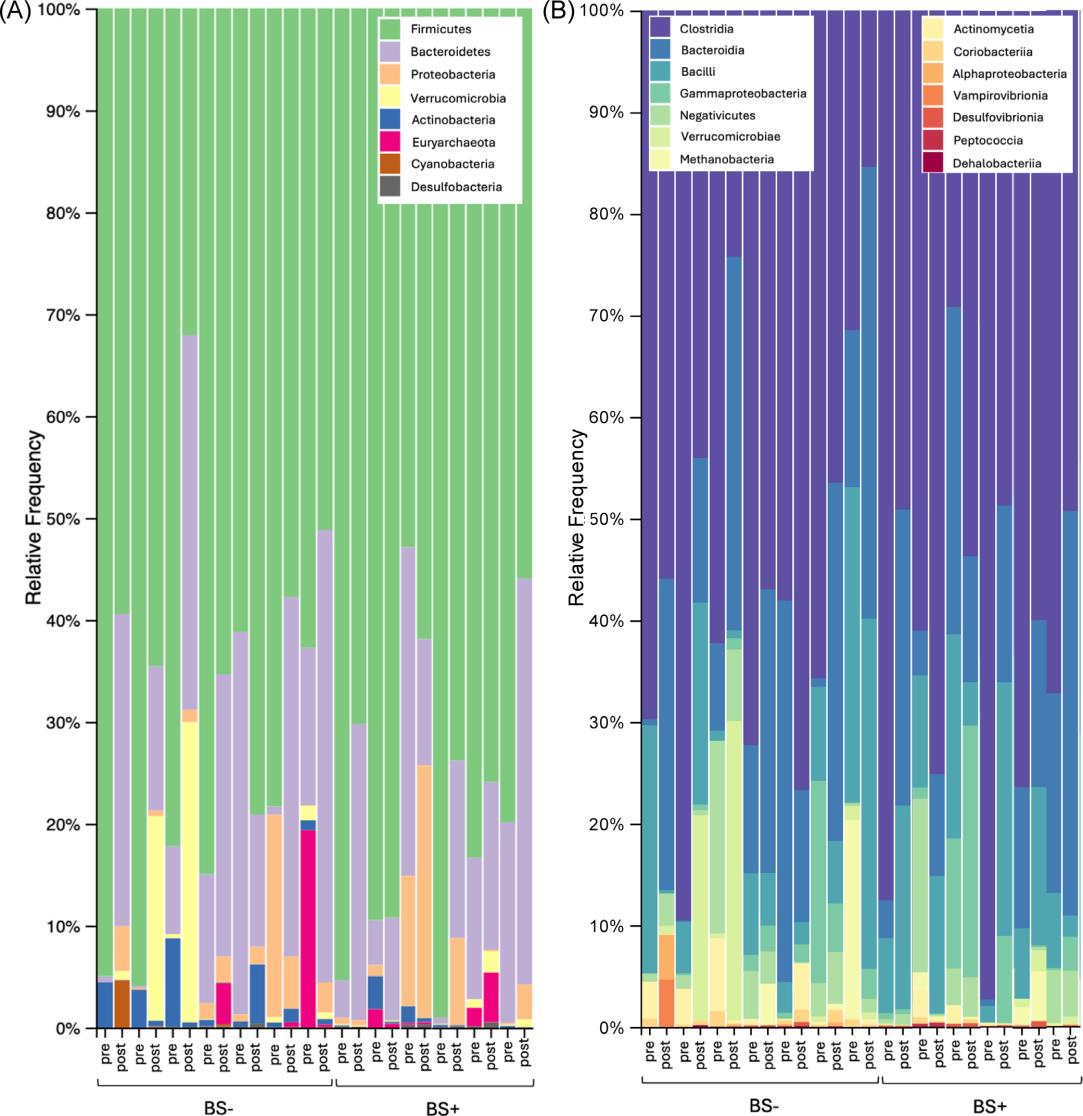
Changes in GM taxonomy over time. (A) Relative frequency of bacterial phyla in the BS‐ and BS+ groups before (pre) and after (post). (B) Relative frequency of bacterial classes in the BS‐ and BS+ groups before (pre) and after (post).

The microbiota phylum composition was different across groups at baseline: the BS+ group exhibited higher relative abundances of Firmicutes and Bacteroidetes, while Verrucomicrobiota, Euryarchaeota and Actinobacteria were lower, although much less represented overall (Figure 1A). Similar differences were seen at class level, the BS+ group showing higher relative abundances of Clostridia, while the BS‐ group exhibiting a greater relative presence of Bacteroidia, Bacilli and Verrucomicrobiae (Figure 1B). At the phylum level, Verrucomicrobia showed the largest increase in BS‐ (log fold change [lfc] = 4.336) compared to a moderate increase in BS+ (lfc = 1.050), with a difference of 3.286. Proteobacteria exhibited a significant increase in BS‐ (lfc = 2.059) versus BS+ (lfc = 0.946), while Actinobacteria decreased markedly in BS+ (lfc = −1.969) but increased in BS‐ (lfc = 0.536), resulting in a difference of 2.505. At the genus level, Akkermansia increased significantly in BS‐ (lfc = 4.336) compared to BS+ (lfc = 1.050), while Sutterella (lfc = 3.636 in BS‐ vs. 1.169 in BS+) and Megamonas (lfc = 4.867 in BS‐ vs. −1.353 in BS+) followed similar trends. Opposite patterns were observed for Streptococcus, which decreased in BS‐ (lfc −1.503) but increased in BS+ (lfc = 0.360), and Bifidobacterium, which increased in BS+ (lfc = −2.334) but showed minimal changes in BS‐. At the species level, *Streptococcus mutans* increased in BS‐ (lfc = 3.417) but decreased in BS+ (lfc = −1.152), whereas *Bacteroides xylanisolvens* showed a marked increase in BS+ (lfc = 4.300).

**FIGURE 2:**
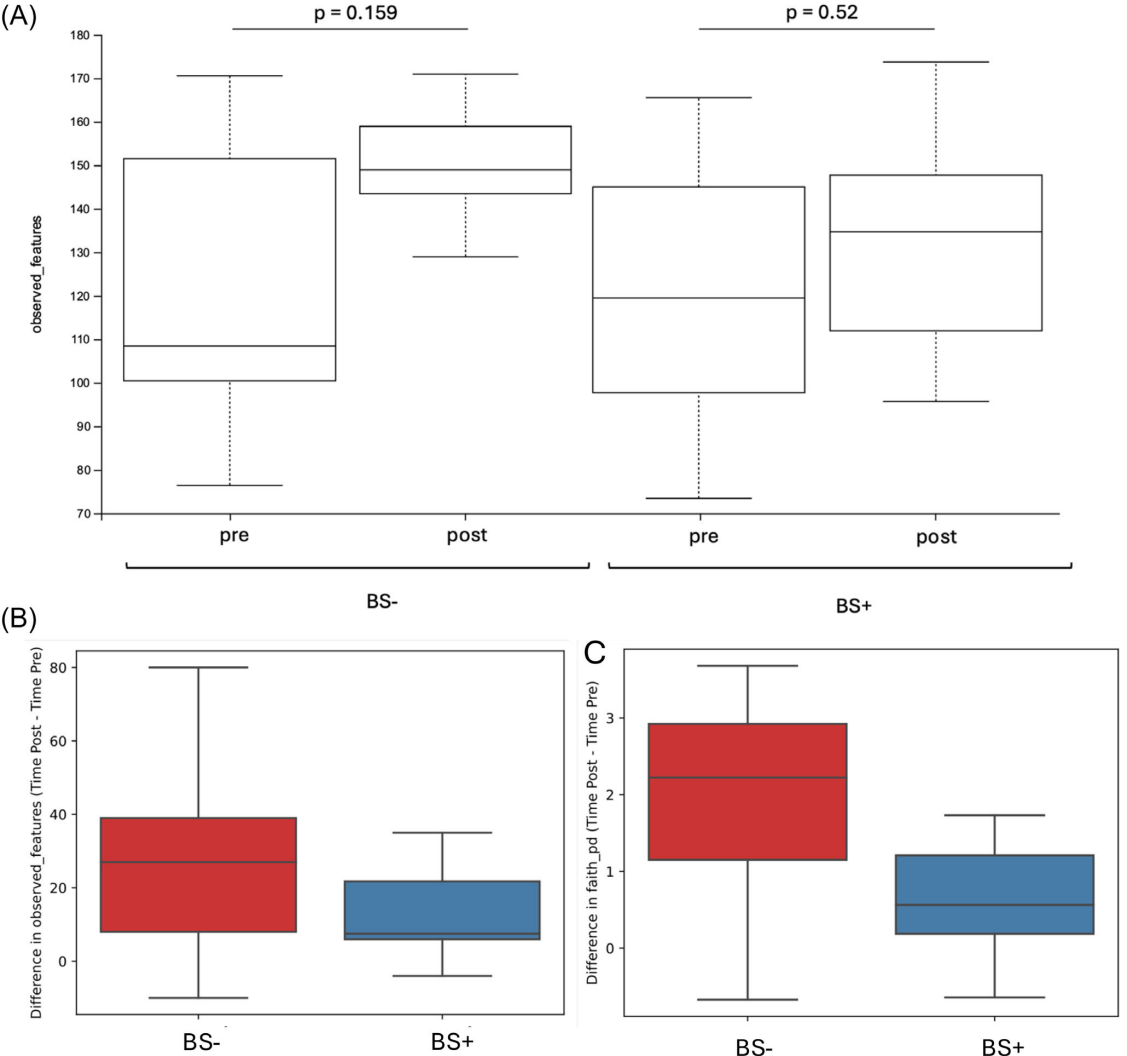
(A) Observed features index for alpha diversity in BS‐ and BS+ groups, comparing pre‐ and post‐intervention time points. (B) Differences in Observed Features Index between post‐ and pre‐intervention timepoints for BS‐ and BS+ groups. (C) Faith phylogenetic diversity (Faith PD) index for alpha diversity.

**FIGURE 3:**
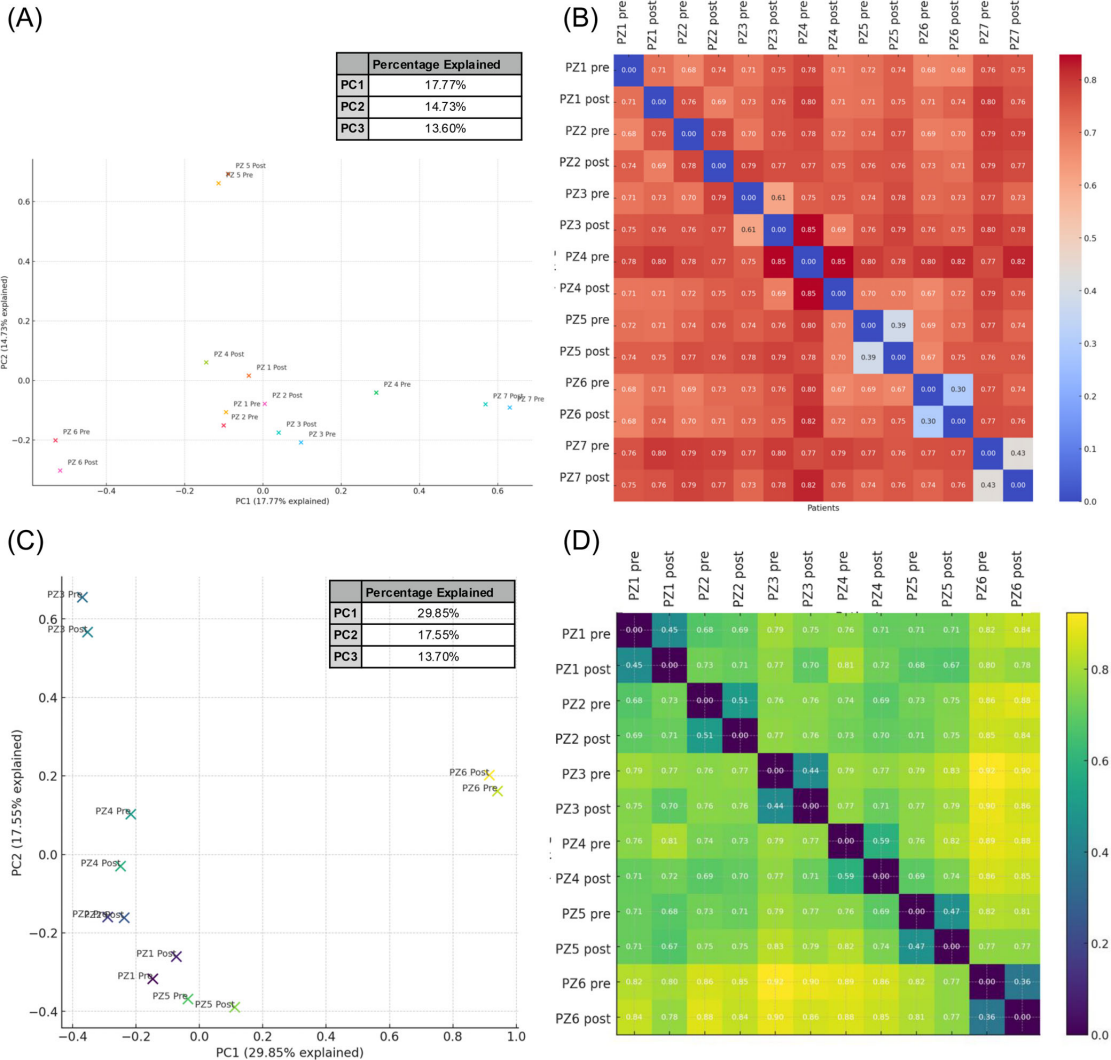
BS‐ group Jaccard Matrix PCA Visualization (A) and Heatmap (B) and BS+ Group Jaccard Matrix PCA Visualization (C) and Heatmap (D) pre‐ and post‐VLCKD Administration.

Alpha diversity analysis, measured through the observed features index, showed that the two groups at baseline were comparable. When analysing changes within groups over time, alpha diversity increased in both, without reaching statistical significance (BS+ p = 0.159; BS‐ *p* = 0.52). However, when accounting for both groups together using a correction for multiple comparisons, the overall increase in alpha diversity over time approached significance (*p* = 0.062) (Figure 2A). In contrast, there was no significant difference in the magnitude of changes between the two groups (*p* = 0.35) (Figure 2B). Faith's Phylogenetic Diversity, which also considers the evolutionary distance between species, revealed a significant increase in the BS‐ group (*p* = 0.03) but not in the BS+ group (p = 0.21), with no difference between the groups (*p* = 0.08) (Figure 2C). Regarding beta diversity, no significant differences were observed across groups over time. For the BS‐ group, a within group comparison was conducted using PERMANOVA with 14 samples (7 per timepoint) and 999 permutations. The analysis yielded a pseudo‐F value of 1.1098, indicating marginally greater variance between timepoints compared to within‐timepoint variance. However, the *p*‐value (0.309) and *q*‐value (0.4414) confirmed no statistically significant differences (Figure 3A,B). Similarly, for the BS+ group, the analysis included 12 samples (6 per timepoint) and 999 permutations, resulting in a pseudo‐F value of 0.4754. This indicated relatively low variance between the timepoints compared to within‐timepoint variance. The *p*‐value (0.932) and *q*‐value (0.968) also confirmed no statistically significant differences (Figure 3C,D).

## 4 DISCUSSION

Our study found that both BS‐ and BS+ groups experienced similar weight loss, and most metabolic parameters were significantly improved, consistent with previous research showing the effectiveness of VLCKD. Mild adverse events were reported in both groups, supporting previous findings on the safety profile of VLCKD.^24^, ^25^

Noteworthy, if BMI was reduced similarly in both groups, smaller improvements were recorded in glucose metabolism in the BS+ group. In prior studies, post‐bariatric patients experienced a significant reduction in fasting glucose, HbA1c and insulin following a VLCKD.^24^, ^25^ The reason why our findings differ from previous studies is unclear, and given overall small sample sizes, it is prudent to exercise caution when interpreting these outcomes. Indeed, BS induces long‐term alterations in gut anatomy, GM and incretin dynamics, which may reduce the additional benefits of dietary interventions such as VLCKD. Additionally, post‐surgery changes in nutrient absorption and metabolic adaptation might attenuate VLCKD's effects. As for the smaller improvement in blood lipids, the same applies, and a slightly worse metabolic health at baseline in patients with no history of BS (i.e. triglycerides were significantly higher at baseline) could be further contributing.

An intriguing finding was that only 38.5% of patients in the post‐bariatric group reached ketosis, compared to 64.7% of bariatric naïve ones. Although this did not prove significantly different, it warrants attention, as this may suggest either reduced compliance in patients with a history of BS and WR or underlying metabolic differences.

Interestingly, eGFR was marginally reduced in BS+ only. Although this was not clinically significant, with small changes within the same kidney function class, it may suggest that post‐bariatric patients kidney function is more sensitive to this dietary regimen. The observed increase in creatinine levels could also be linked to the metabolic and physiological adaptations in post‐BS patients combined with the protein‐rich composition of the VLCKD: following BS, patients often struggle to achieve the recommended protein intake due to reduced gastric capacity, altered taste preferences and food aversions. While protein digestion and absorption remain largely intact after surgery, a sudden transition to a VLCKD, inherently relatively high in protein, may affect renal function in patients with prior low protein consumption. However, no evidence is available in support, warranting ad hoc studies. To prevent kidney strain, proper hydration of at least 2 L/day is essential, and incorporating plant‐based protein sources can help reduce the renal burden while meeting dietary protein requirements effectively.^30^

Diets shape GM composition,^31^ yet the effect of a VLCKD, particularly in post‐BS patients, remains largely underexplored.^23^, ^32^ Our results revealed that despite comparable alpha diversity at baseline, the GM composition differed significantly across groups, likely due to BS lasting effects on the GM. Notably, the BS+ group exhibited lower relative abundances of Actinobacteria, consistent with previous findings reporting a decrease in females but an increase in males after RYGB surgery over a six‐year follow‐up.^33^ Unexpectedly, the BS+ patients at baseline also showed lower levels of Verrucomicrobia. This is surprising, as other studies often report a short ‐term increase.^34^, ^35^ The high time variability between BS and sample collection could explain these discrepancies and introduces a potential bias in interpreting GM shifts post‐surgery. Alpha diversity changes following VLCKD were more pronounced in the BS‐ group, as evidenced by the significant increase in Faith's Phylogenetic Diversity, whereas the BS+ group exhibited a non‐significant increase. This finding aligns with the observation that BS+ patients displayed a less dynamic response in microbial diversity metrics, likely reflecting a post‐surgical stabilization of the GM. In our study, beta diversity analyses supported these observations, as no significant differences were detected, suggesting that, while the VLCKD influenced GM composition, it did not lead to major shifts in overall community structure. The changes in bacterial strains observed indicate that the VLCKD positively influenced GM composition in both groups, with a more pronounced impact in BS‐ compared to BS+. Despite WR, the BS+ group's dysbiosis appeared less severe, likely reflecting the lasting positive effects of BS on the GM.

Our study has several limitations. Its single‐centre design and short duration may restrict the generalizability of findings and the ability to assess long‐term effects. The small sample size, yet a priori calculated, and variability in the intervals between surgery and sample collection, as well as a broad WR range, limit the potential for subgroup analyses. The inclusion of patients undergoing two types of BS also introduced heterogeneity, as RYGB and VSG have distinct effects on GM composition. Moreover, the absence of pre‐surgery faecal samples in the BS+ group hindered the assessment of GM changes directly attributable to BS over time. Animal models might address some limitations, but their translational relevance is constrained by differences in gluconeogenic capacity between humans and mice,^36^ while expanding the sample size and extending the follow‐up would instead better strengthen our findings. Lastly, the study's focus on comparing the safety and efficacy of a VLCKD in BS+ patients versus BS‐ individuals did not allow for direct comparisons between VLCKD and other dietary interventions for addressing post‐BS WR. Future research should evaluate VLCKD against alternative dietary approaches to better define its relative effectiveness in this population.

Our study also features some strengths. This is, to the best of our knowledge, the first study to evaluate the differential effects of a VLCKD between post‐bariatric patients experiencing WR and bariatric‐naïve individuals, providing insights into the potential differential impacts of this dietary approach. Cases and controls were matched for age, sex and BMI, allowing for more accurate comparisons. Through regular dietary recalls and objective biomarkers, our study ensured robust evaluation of dietary adherence. Finally, GM analysis allows for an exploration of the potential pathways driving the observed effects of VLCKD.

The study's findings may have practical implications, supporting the use of VLCKD as a treatment option for both pre‐ and post‐bariatric patients. The potentially smaller metabolic improvements observed in the BS+ group underscore the importance of tailored patient education and possibly stricter dietary parameters, such as slightly reducing calorie and carbohydrate intake and focusing on very low glycaemic index vegetables in order to maximize its beneficial effects, always in keeping with current recommendations.^18^ However, the scarcity of data on VLCKD in post‐bariatric WR highlights the need for further studies to confirm our findings.

In conclusion, our study highlights the potential of a VLCKD in managing post‐bariatric weight regain (WR), emphasizing the need for careful monitoring of kidney function to prevent adverse effects.

## CONFLICT OF INTEREST STATEMENT

The authors declare no competing interests.

### PEER REVIEW

The peer review history for this article is available at https://www.webofscience.com/api/gateway/wos/peer‐review/10.1111/dom.16187.

## Data Availability

Data available upon reasonable request to the corresponding author.
